# DFT Study of Molecular and Electronic Structure of Y, La and Lu Complexes with Porphyrazine and Tetrakis(1,2,5-thiadiazole)porphyrazine

**DOI:** 10.3390/molecules26010113

**Published:** 2020-12-29

**Authors:** Yuriy A. Zhabanov, Igor V. Ryzhov, Ilya A. Kuzmin, Alexey V. Eroshin, Pavel A. Stuzhin

**Affiliations:** Research Institute of Chemistry of Macroheterocyclic Compounds, Ivanovo State University of Chemistry and Technology, Sheremetievskiy av. 7, 153000 Ivanovo, Russia; ryzhoff.ihor@yandex.ru (I.V.R.); wonderful_37@list.ru (I.A.K.); alexey.yeroshin@gmail.com (A.V.E.); stuzhin@isuct.ru (P.A.S.)

**Keywords:** porphyrazine, 1,2,5-thiadiazole annulated, DFT study, molecular and electronic structure

## Abstract

**Abstract:**

Electronic and geometric structures of Y, La and Lu complexes with porphyrazine (Pz) and tetrakis(1,2,5-thiadiazole)porphyrazine (TTDPz) were investigated by density functional theory (DFT) calculations and compared. The nature of the bonds between metal atoms and nitrogen atoms has been described using the analysis of the electron density distribution in the frame of Bader’s quantum theory of atoms in molecule (QTAIM). Simulation and interpretation of electronic spectra were performed with use of time-dependent density functional theory (TDDFT) calculations. Description of calculated IR spectra was carried out based on the analysis of the distribution of the potential energy of normal vibrations by natural vibrational coordinates.

**Sample Availability:**

Not available.

## 1. Introduction

Macroheterocycles such as porphyrines, phthalocyanines and their analogues have found a number of applications [1,2,3,4]. The possibility of modification of peripheral substituents [5] or atoms in a central ring allows fine-tuning or modulation of their properties [5,6] such as stability, rigidity, extended conjugated π-electron cloud, conductivity, light absorption capability, long-lived fluorescence, possibility to host uncoupled electrons on the central metal ion or in the aromatic cloud, and so on [7].

Substitution of benzene rings in phthalocyanines by aromatic heterocycles, e.g., by pyrazine [8,9] or 1,2,5-chalcogenadiazole [10] have a strong impact of the electronic properties of the central porphyrazine (Pz) core which is common for these systems.

The presence of a five-membered heterocycle containing nitrogen and sulfur atoms on the periphery of tetrakis(1,2,5-thiadiazole)porphyrazine (TTDPz) considerably modulates the physico-chemical properties of the macrocycle and its ability for intermolecular interaction as compared to phthalocyanines [10]. Unlike phthalocyanines, or their pyrazine-fused analogues, TTDPz have no H atoms on the periphery and their molecular packing during crystal growth is determined by specific N…S interactions [11]. This is quite important for application of these phthalocyanine-type molecules as building blocks for novel functional materials in various fields [12].

Lanthanide complexes of phthalocyanines due to their rich structural diversity and peculiar spectral and electrochemical properties [13] are especially interesting as components of different devices [14]. Among the metal complexes of TTDPz, macrocycle complexes of p- and d-metals are known [MTTDPz]: M = Li [15], Mg [16], Ca [17], AlCl, GaCl, InOAc [18], Zn, Cu, Ni, Co, Fe, Mn [11,16,19]. Recently we have also obtained first representatives of the rare earth metal complexes [(acac)YTTDPz], [(acac)LuTTDPz] [20]. Among lanthanide complexes of unsubstituted porphyrazine [(acac)MPz] complexes with dysprosium, neodium and europium have been reported [21,22].

The optical and coordination properties of phthalocyanine-type complexes crucially depend on the electronic structure of the metal and macrocycle and can often be predicted by quantum-chemical calculations. Such investigations in the case of transition metal complexes s are often nontrivial due to the necessity to account for the multireference character of the wavefunction [23]. However the information about the ground-state properties can be readily obtained for closed-shell species. Density functional theory (DFT) provides a straightforward way to obtain quantitative and qualitative information about structural, electronic and spectral features. It is quite important to investigate the influence of a transition metal and a macrocyclic ligand on the properties of the porphyrine-type complexes, particularly, the peculiarities of chemical bonding and spectral properties. Comparison in the series of atoms of d^1^ metals with a different radius (Y, La and Lu) allows the taking into account of only the size of atoms. The change of the periphery of the porphyrazine macrocycle (Pz) by fusion of electron-deficient 1,2,5-thiadiazole rings (TTDPz) allows the determination of the influence of the ligand on the coordination properties of central atoms.

Earlier in our laboratory, the Ca, Fe, Co and Zn complexes with porphyrazines and tetrathiadiazoporphyrazines ligands were investigated by quantum-chemical calculations [17,23]. Preliminary quantum-chemical calculations and interpretation of vibrational spectra were also carried out for complexes of tetra(1,2,5-thiadiazolo)porphyrazine with rare earth elements Y and Lu [20]. The main objective of the present study is to identify the influence of the molecular and electronic structures on the properties of macrocyclic complexes. It is quite important to compare the TTDPz complexes with the corresponding complexes of nonsubstituted porphyrazine in order to reveal the impact of annulation of the 1,2,5-thiadiazole rings. The nature of the chemical bonding between metal atoms and nitrogen atoms has been described using the analysis of the electron density distribution in the framework of Bader’s quantum theory of atoms in molecules (QTAIM) [24]. The lowest excited states were also calculated in order to explain the peculiarities and tendencies observed in the experimental electronic absorption spectra available for the Y and Lu tetra(1,2,5-thiadiazolo)porphyrazine complexes [20]. In addition, the vibrational absorption spectra were analyzed and interpreted.

## 2. Results and Discussion

### 2.1. Geometric Structure

**Figure 1:**
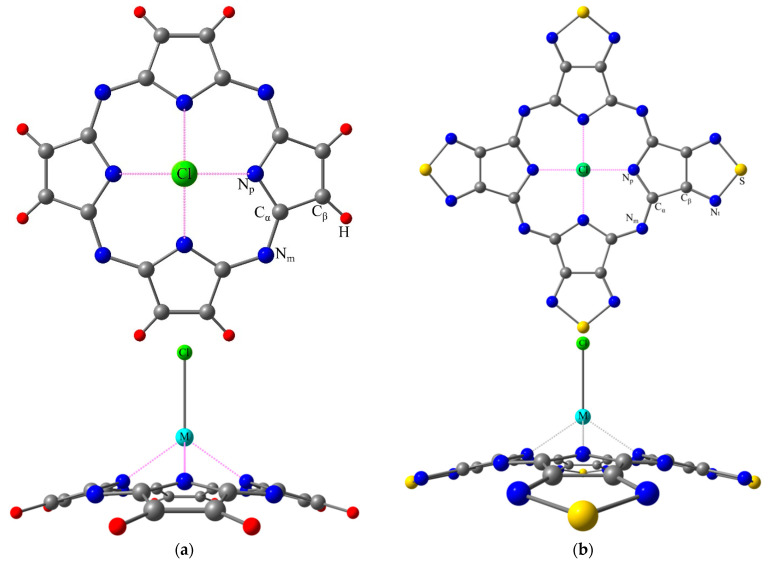
Molecular models of porphyrazine (MClPz) (**a**) and tetra(1,2,5-thiadiazolo)porphyrazine (MClTTDPz) (**b**) complexes with atom labeling (M = Y, La, Lu).

Equilibrium structures of the Y, La and Lu macroheterocyclic complexes with porphyrazine (MClPz) and tetra(1,2,5-thiadiazolo)porphyrazine (MClTTDPz) bearing chloride as an axial ligand, were found to possess C_4v_ symmetry with doming distortion (Figure 1). Force-field calculations yielded no imaginary frequencies, indicating that the optimized configurations correspond to the minima on the potential energy hypersurfaces. The calculated molecular parameters are listed in Table 1.

Analysis of the data in Table 1 shows that there is a significant difference (maximum is about 0.2 Å) in the internuclear distances M-Cl and M-N_p_ in the series of studied complexes, as well as in the parameters M-X1 and X1-X2 which characterize the distortion of the macrocyclic ligand. The change in the size of the coordination cavity (N_p_…N_p_)_opp_ of the ligand is also quite large in MClTTDPz over against MClPz. Additionally there is an increase of M-Cl, M-N_p_, (N_p_…N_p_)_opp_ and (N_p_…N_p_)_adj_ distances in the LuClPz-YClPz-LaClPz series, corresponding to the increase in the ionic radius of metals [25]. A similar situation is observed for MClTTDPz. It should be noted that changes in the analogous parameters of MPz and MTTDPz complexes with a number of transition, alkali and alkali earth metals [15,17,23] are comparable to changes in the parameters of the complexes considered in this work. Obviously, such differences are due to the nature of the metals introduced into the macrocycle cavity. However, the nature of the metal has practically no effect on the structure of the periphery of the ligand, since the internuclear distances N_p_-C_α_, C_α_-N_m_, C_α_-C_β_, C_β_-C_β_, C_β_-H (for MClPz), C_β_-N_t_, and N_t_-S (for MClTTDPz) in the macrocyclic fragment change by no more than 0.002 Å, and the bond angles N_p_C_α_N_m_, C_α_N_m_C_α_, C_α_N_p_C_α_ and N_t_SN_t_ (for MClTTDPz) change by no more than 0.5 degrees. Thus, we can conclude that the change in metal affects only the size of the macrocyclic cavity. It should also be noted that the introduction of thiadiazole as a substituent changes the bond lengths of pyrrole moieties by no more than 0.07 Å, and the values of bond angles change by no more than 4 degrees.

The value of lanthanide contraction Δr_Ln_, which may be estimated as a difference between r(La-N_p_) and r(Lu-N_p_) distance values, is 0.202 Å for both MClPz and MClTTDPz. It should be noted that the obtained value of Δr_Ln_ is close to similar theoretical value in the series of compounds with tridentate ligand (0.208 Å) [26], experimental values in the series of ML_3_ with monovalent ligands, such as the series of trichlorides (0.186(8) Å), tribromides (0.185(8) Å) and triiodides (0.193(8) Å) [27,28,29,30,31], as well as in the series of compounds with bidentate ligands, for example, in the series of tris-dipivaloylmethanates (0.186(6) Å) [32,33]. The similarity of Δr_Ln_ values in lanthanide compounds with three ligands and in complexes considered in the present study indicates that, with no regard of the size of the central atom in the lanthanide series, the geometric structure of the coordination cavity is adjusted to the size of the central atom with the formation of coordination bonds.

### 2.2. Electron Density Distribution and Chemical Bonding in MClPz and MClTTDPz

Selected net atomic charges and topological parameters of the electron density in bond critical points (BCP) are listed in Table 2. According to the Table 2 the incorporation of thiadiazole rings does not lead to a significant change in the charge of the metal and the ligands (the charge of the macroheterocyclic ligand is calculated as the sum of the net atomic charges on the ligand atoms). The presence of electron-withdrawing nitrogen atoms in the thiadiazole rings induces a shift of electron density from the central cavity and leads to a charge transfer in the row N_t_←C_β_←C_α_. It should be noted that the charges on the atoms of the internal macrocycle (C_α_, N_p_, N_m_) also do not change significantly, but the charge on the carbon atom C_β_ increases by 0.4 e (Table 2). Similar tendencies in the change of the charges were revealed in the complexes MPz and MTTDPz with transition metals Ca, Fe, Co and Zn [17,23]. The charge on the metal atoms is about +2.2 e, which is comparable to the charge on the Y, La and Lu atoms in the tridentate macroheterocyclic ligand [26]. In the studied complexes the charge of a ligand varies from −1.447 to −1.362 e, and in complexes with transition metals from −1.580 to −1.141 e. In addition, the charges on the atoms of the internal macrocycle (C_α_, N_p_, N_m_) are close to each other in the case of all the complexes considered (the complexes studied in this work and the complexes with transition metals [17,23]). Based on the above-mentioned considerations, it can be concluded that the electron density distribution in the coordination cavity of metal complexes of porphyrazine and tetra(1,2,5-thiadiazole)porphyrazine metal does not change significantly regardless of metal nature and thiadiazole annelation.

The nature of a chemical bond can be determined by the value of the electron density, laplacian ∇^2^ρ. A positive value of the electron density laplacian ∇^2^ρ indicates ionic interaction. However, the positive values of ∇^2^ρ for M-Cl bonds, as well as the corresponding delocalization indices δ(M|Cl) representing the magnitudes of the electron exchange between the basins of the corresponding atoms, allow to argue that these bonds, along with an ionic component (Table 2), possess a significant covalent component (about 0.6). Moreover, it can be noted that the internuclear distances r(M-Cl) decrease by 0.03 Å and the delocalization indices increase by about 0.04 in the MClTTDPz complexes as compared to their MClPz analogues. Values of the parameters ∇^2^ρ, δ(M|N_p_) and q(M|N_p_) (Table 2) indicate mainly the ionic M-N_p_ bond with a noticeable covalent component (value of δ(M|N_p_)) comparable to the corresponding value for the Pz and TTDPz complexes with Ca and Zn [17].

### 2.3. Electronic Absorption Spectra

**Figure 2:**
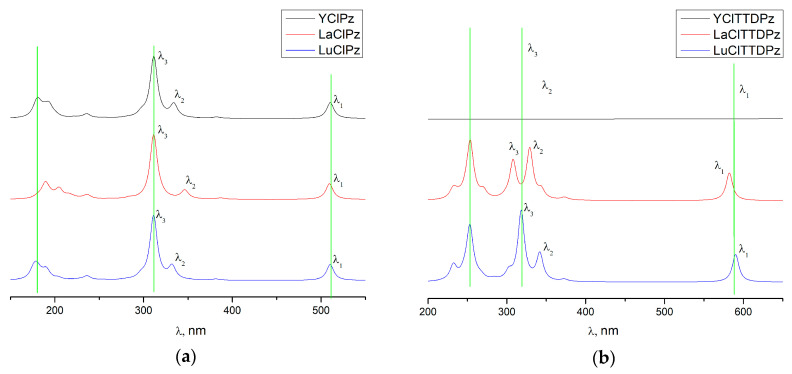
Calculated time-dependent density functional theory (TDDFT) electronic absorption spectra for MClPz (**a**) and MClTTDPz (**b**) complexes.

The electronic absorption spectra of MClPz and MClTTDPz molecules simulated on the basis of time-dependent density functional theory (TDDFT) calculations are shown in Figure 2. The calculated electronic spectra of complexes MClPz and MClTTDPz (M=Y, La, Lu) are quite similar. The main difference in the electronic absorption spectra in the MClPz series is the bathochromic shift of the B_x_ (λ_2_) band. In the case of MClTTDPz molecules, the difference in the electronic spectra appears in the change of positions and relative intensities of the B_x_ (λ_2_) and B_y_ (λ_3_) bands, as well as in the relatively small bathochromic shift of the Q-band (λ_1_). In general, the spectra can be described by the model of Gouterman [34,35,36], who suggested electronic absorption spectra of porphyrazine molecules, to possess four intense bands B_x_, B_y_, Q_x_, and Q_y_, and three bands (B_x_, B_y_, and Q) in the case of their metal complexes. Interpretation of the electronic spectra was carried out on the basis of the results of TDDFT calculations. The calculated oscillator strengths (f) for the lowest excited states along with their composition (in terms of one-electron transitions) are given in Table 3 (full list of the most active transitions can be found in Supplementary Materials). Analysis of the data in Table 3 demonstrates that there are three excited electronic states of MClPz molecules characterized by a high oscillator strength. These transitions correspond to three intense bands—B_x_, B_y_, and Q. The low-lying calculated excited state 1^1^E for MClPz complexes is formed predominantly by the electronic transitions from HOMO and HOMO-1 to LUMO, which can be assigned to the Q-band. It should be noted that the compositions of the wave functions and the oscillator strengths corresponding to the electronic state 1^1^E are very close to each other for all MClPz (M = Y, La, Lu) complexes. The excited state 4^1^E of MClPz is formed by the similar electronic transitions and corresponds to the B_x_ band, which is shifted to larger wavelengths in the case of LaClPz. Transitions in the higher excited states are predicted to have a highest oscillator strength and can be associated with the B_y_ band. These excited states (6^1^E for LaClPz and 5^1^E for YClPz and LuClPz complexes) are composed mainly of the electronic transitions from a_1_ and a_2_ orbitals to the LUMO e*. The changes of electronic spectra are more significant in complexes of the MClTTDPz (Figure 2, Table 3), where the B_x_ and B_y_ bands are different in wavelengths and relative intensities. Thus, it can be concluded that the incorporation of a substituent can enhance the effect of the metal on the spectral properties of the complexes.

### 2.4. Molecular Orbitals

**Figure 3:**
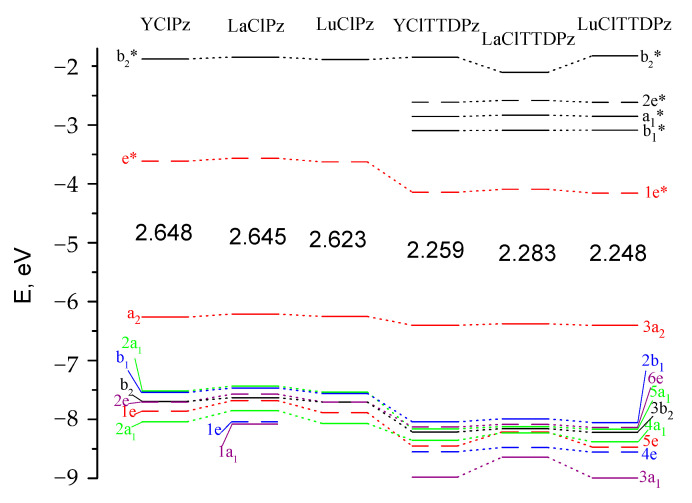
Molecular orbital (MO) level diagram for MClPz and MClTTDPz complexes. The values of highest occupied molecular orbital-lowest unoccupied molecular orbital (HOMO-LUMO) gaps are given in eV.

The energy diagram of molecular orbitals for MClPz and MClTTDPz is shown in Figure 3. The HOMO energies are close in the series LaClPz-YClPz-LuClPz and LaClTTDPz-YClTTDPz-LuClTTDPz, which allows us to conclude that the influence of a metal is insignificant. At the same time, the fusion of thiadiazole rings to the porphyrazine macrocycle leads to a decrease in the HOMO energy, which indicates an increase in the acceptor properties of the compound. In addition, the thiadiazole ring presence significantly reduces the HOMO–LUMO gap from approximately 2.6 to 2.3 eV. A similar situation was observed when considering the Ca(II) and Zn(II) complexes with porphyrazine and tetrakis(1,2,5-thiadiazole)porphyrazine [17] and the HOMO–LUMO gap of magnesium(II) tetra(1,2,5-chalcogenadiazolo)porphyrazines, [TXDPzMg] (X=O, S, Se, Te) is close and varies from ~2.0 to ~2.2 eV [37].

**Figure 4:**
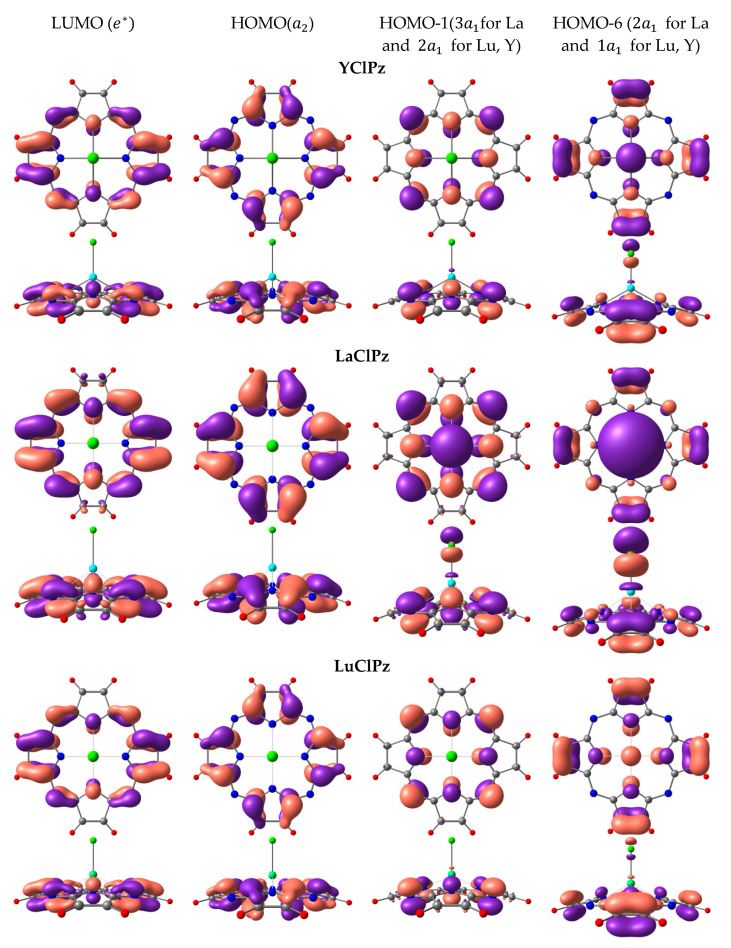
Influence of the metal (Y, La, Lu) on the molecular orbitals of MClPz complexes.

**Figure 5:**
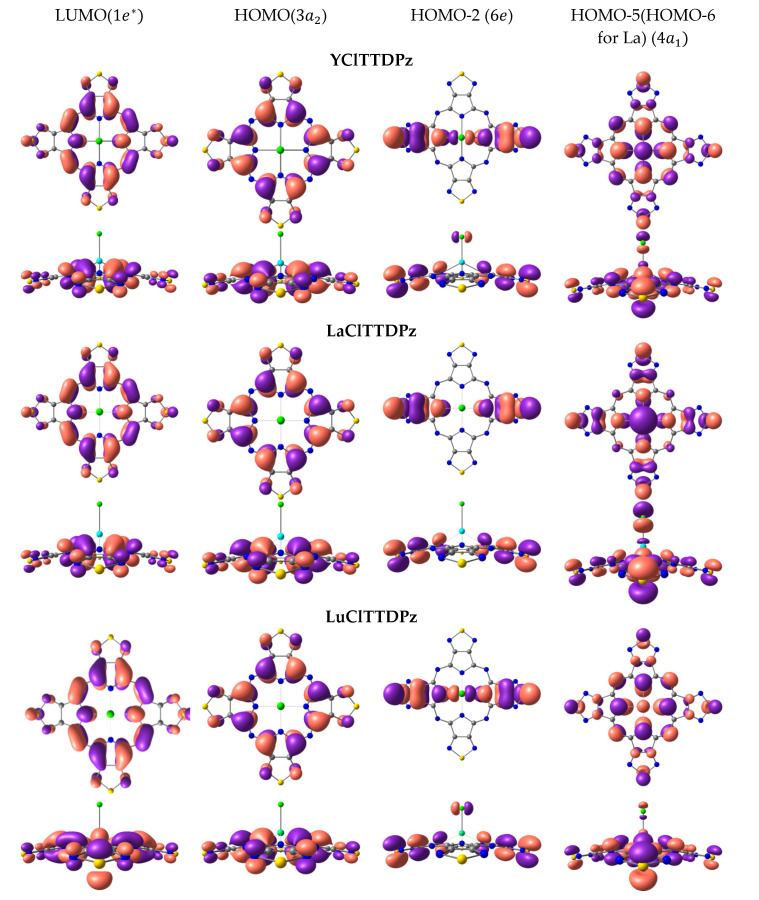
Influence of the metal (Y, La, Lu) on the molecular orbitals of MClTTDPz complexes.

The shapes of molecular orbitals (MOs) that participate in electronic transitions with large oscillator strengths are shown in Figure 4 and Figure 5. Figure 4 and Figure 5 show that LUMO and HOMO are linear combinations of π-bonding and π-antibonding molecular orbitals of the macrocycle, while HOMO-1 and HOMO-6 contain both atomic orbitals (AOs) of the macrocycle and AOs of metal and axial ligand Cl. It should be noted that the shapes of HOMO and LUMO for all considered complexes are similar. However, HOMO exhibits a bonding character for the C_α_-C_β_ bond in the case of MClPz and an antibonding character for MClTTDPz complexes. Differences in the molecular orbitals of the complexes MClPz are observed in the compositions of the HOMO-1 and HOMO-6 MOs (Figure 4). Thus, in the LaClPz molecule, the electron density is shifted towards the Cl atom, while in LuClPz and YClPz the Cl atom has practically no electron density (HOMO-1). In MClTTDPz molecules, the main contribution to transitions to excited electronic states with the highest oscillator strength is made by the LUMO, HOMO, HOMO-2, HOMO-3, and HOMO-5 (HOMO-4 for LaClTTDPz) orbitals. The shapes of these orbitals are shown in Figure 5. A comparative analysis of the selected MOs reveals differences in the shape of 6e orbitals. In the case of LaClTTDPz, the electron density belongs to the macrocycle, while in the La and Y complexes, the orbitals of the chlorine atom contribute. Of further note is the shape of the 4a_1_ orbitals, which are a linear combination of bonding and antibonding MOs of the macrocycle and metal. Thus, it can be observed that, in the LaClTTDPz molecule, it includes a bonding π-orbital at the C_β_-C_β_ bond, while these MOs of the LuClTTDPz and YClTTDPz molecules can be considered as completely antibonding.

### 2.5. Vibrational Spectra

**Figure 6:**
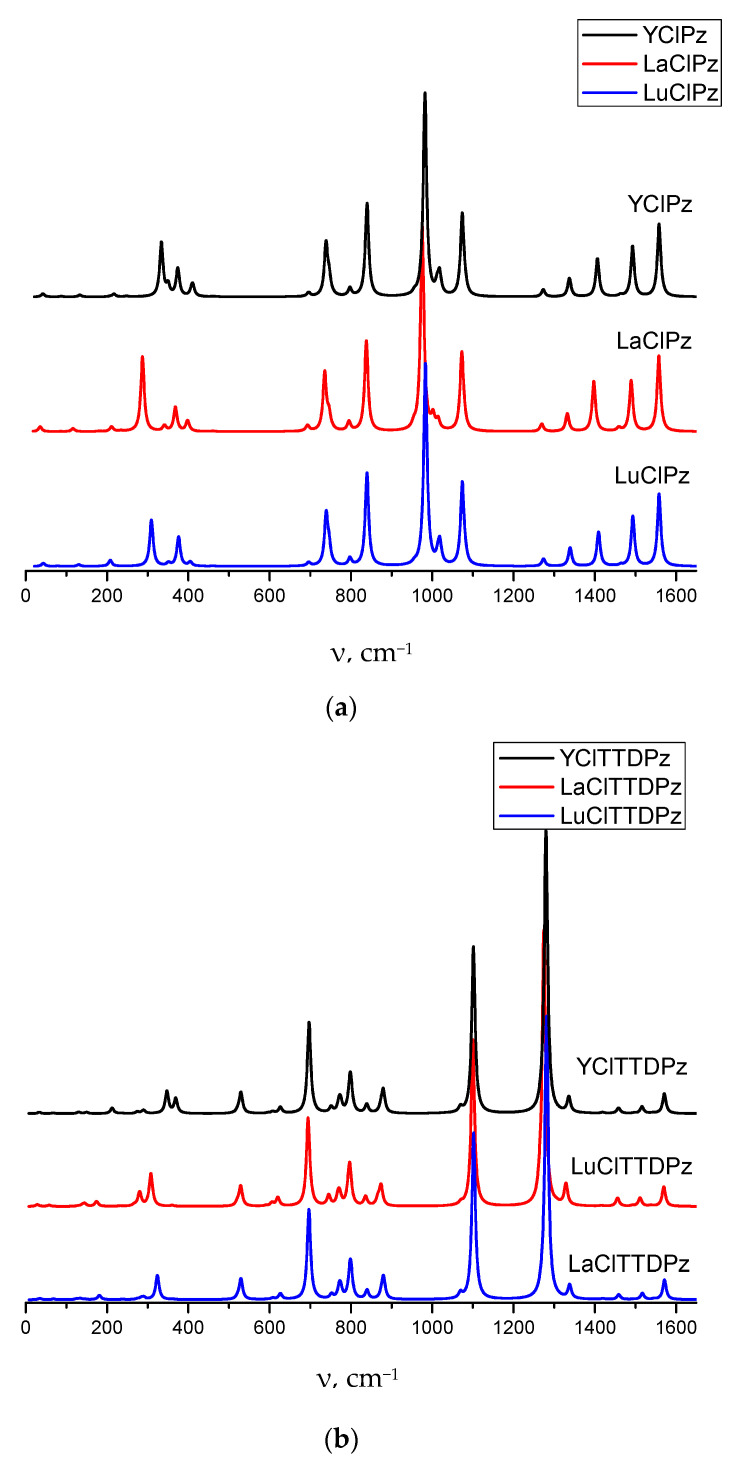
Simulated vibrational spectra of the MClPz (**a**) and MClTTDPz (**b**) complexes.

Recently, a description of the absorption bands of the vibrational spectrum of YClTTDPz and LuClTTDPz complexes was published in [20]. Detailed interpretation of IR-spectra for MClTTDPz and MClPz is performed in the present work. The IR spectra were simulated on the basis of the normal mode frequencies and band intensities, which have been calculated by the DFT (B3LYP/pcseg-2) method in a harmonic approximation. Description of the main vibrations is presented in Table 4 (full list of the most active vibrations can be found in Supplementary Materials) and simulated spectra are shown in Figure 6.

In the Y-La-Lu series a high-frequency band shift occurs both in MClTTDPz and in MClPz. The main differences between MClTTDPz and MClPz spectra occur in the positions and characters of the most intensive band, as well as the larger number of intense peaks in MClPz especially noticeable in high-frequency region. The most intensive peak is located in the 982 cm^−1^ region and corresponds predominantly to C_α_-C_β_ stretching for MClPz complexes. In the case of MClTTDPz the strongest band is observed at ~1290 cm^−1^ (ω_87_-ω_88_) and mainly represented by stretching and bending in the macrocyclic fragment. Spectra of MClPz contain more intensive peaks as compared to MClTTDPz. The largest contribution to the ω_85_-ω_86_ bands in MClPz is C_β_-C_β_ stretching. The N_m_-C_α_ stretching vibrations contribute strongly to relatively weak bands ω_82_-ω_83_. In the case of the LaClPz ω_76_ band representing mainly in-plane bending/stretching, it possesses a higher intensity than the corresponding one in LuClPz and YClPz. The out-of-plane deformations provide a major contribution to the medium band ω_48_. The M-Cl stretching vibrations make a significant contribution to the weak bands in the low frequencies region for MClPz and for MClTTDPz too. MClTTDPz have similar IR-spectra in the 500–1600 cm^−1^ region where the influence of the metal is almost absent. In this region vibrations of the macrocyclic core are observed. The ω_50_-ω_51_ and ω_80_-ω_81_ bands in MClTTDPz spectra are composed by N_p_-C_α_ and N_m_-C_α_ stretching vibrations combined with in-plane deformations of the rings. Despite the noted significant covalent character of the bonding of the metal with the macrocycle, the stretching vibrations involving metal are detected only in the low frequencies region or have a low intensity. A performed description of calculated IR spectra shows that the most observed bands cannot be assigned to any single vibrational mode and are the result of the some vibrational modes superpositions.

## 3. Materials and Methods

### Computational Details

The DFT-based investigation of MClPz and MClTTDPz included geometry optimizations and computations of the harmonic vibrations followed by TDDFT calculations of the electronic absorption spectrum. The number of the calculated excited states was 30. The calculations were performed using the B3LYP functional with the use of pcseg-2 basis set [38] taken from the EMSL BSE library [39,40] for describing the electron shells of C, N, S, Cl and H atoms. For describing the 28 core electron shells and 11 valence electrons of the yttrium atom pseudopotential combined with corresponding basis set cc-pVTZ-PP [41] were used. In case of the lanthanum atom 46 core electron shells were described by pseudopotential [42] and 11 valence electrons were described by Def2-TZVPP [42] basis sets. For the description of 11 valence electrons of the Lu atom triple-ζ valence basis sets [43] were used and 60 core shells were described by an effective core pseudopotential [44] developed by the Stuttgart group. The Firefly QC [45] package, which is partially based on the GAMESS(US) [46] source code was used in all the calculations.

Optimized Cartesian coordinates of MPz and MTTDPz are available from Supplementary Materials.

Description of the vibrational modes is carried out based on the analysis of the distribution of the potential energy of normal vibrations by natural vibrational coordinates. This analysis was performed using the VibModule program [47].

The quantum theory of atoms in molecules (QTAIM) analysis [48] was performed using the AIMAll [49] software package. Topological parameters of *ρ(r)* in bond critical points and charges on atoms are collected in Supplementary Materials.

The molecular models and orbitals demonstrated in the paper were visualized by means of the Chemcraft program [50].

## 4. Conclusions

The influence of the nature of the metal (Y, La, Lu) and the ligand (either porphyrazine or thiadiazole-annelated porphyrazine) on the geometry, electronic structure and electronic and vibrational absorption spectra of the macroheterocyclic complex was studied by DFT calculations at the B3LYP/pcseg-2 level.

It has been shown that the ligand cavity can adjust itself to the size of the central atom in a way that results in doming distortion of the ligand without changing the structure of the periphery of the macrocycle. It has been found that the electron density distribution in the coordination cavity of metal complexes of porphyrazine and tetra(1,2,5-thiadiazole)porphyrazine slightly depends on the nature of the ligand and metal.

The value of the lanthanide contraction Δr_Ln_ in the studied complexes has been determined, which turned out to be close to Δr_Ln_ in the series of lanthanide halogenides with three monovalent ligands and in the series of lanthanide compounds with three bidentate ligands.

The incorporation of a substituent can enhance the effect of the metal on the spectral properties of the complexes. The electronic spectra of investigated complexes can be described by the model of Gouterman. It has been shown that in the calculated IR spectra the most intense bands cannot be assigned to any single vibrational mode and are the result of the superposition of vibrational modes.

## Figures and Tables

**Table 1:** Internuclear distances (r_e_, in Å) and valence angles (∠, in deg.) of the equilibrium C_4v_ structures by B3LYP/pcseg-2 calculations.

	LaClPz	LaClTTDPz	LuClPz	LuClTTDPz	YClPz	YClTTDPz
M-Cl	2.711	2.680	2.493	2.468	2.523	2.497
M-N_p_	2.462	2.486	2.260	2.284	2.291	2.313
N_p_-C_α_	1.369	1.377	1.370	1.379	1.370	1.379
C_α_-N_m_	1.330	1.321	1.329	1.319	1.329	1.319
C_α_-C_β_	1.456	1.460	1.454	1.458	1.454	1.458
C_β_-C_β_	1.355	1.423	1.355	1.422	1.355	1.422
C_β_-H	1.077		1.077		1.077	
C_β_-N_t_		1.316		1.317		1.317
N_t_-S		1.644		1.643		1.643
(N_p_…N_p_)_opp_	4.023	4.139	3.979	4.096	3.987	4.101
(N_p_…N_p_)_adj_	2.844	2.927	2.813	2.897	2.819	2.900
∠ (N_p_MCl)	125.2	123.6	118.3	116.3	119.5	117.6
∠ (MN_p_C_α_)	124.7	122.4	125.1	123.1	125.2	122.9
∠ (N_p_C_α_N_m_)	127.6	128.1	127.3	127.9	127.4	128.0
∠ (C_α_N_m_C_α_)	125.0	127.1	124.5	126.6	124.6	126.7
∠ (C_α_N_p_C_α_)	107.5	111.6	107.6	111.5	107.6	111.6
∠ (N_t_SN_t_)		100.3		100.4		100.4
M-X1 ^1^	1.419	1.377	1.072	1.011	1.129	1.071
X1-X2 ^2^		1.028		0.775		0.767

^1^ X1 is dummy atom located in center between N_p_ atoms. ^2^ X2 is dummy atom located in center between S atoms.

**Table 2:** Selected parameters of MClPz and MClTTDPz complexes from quantum theory of atoms in molecules (QTAIM) calculations.

	YClPz	LaClPz	LuClPz	YClTTDPz	LaClTTDPz	LuClTTDPz
∇^2^ρ (M-N_p_), a.u.	0.205	0.160	0.235	0.193	0.152	0.221
q(M|N_p_)	0.348	0.356	0.340	0.354	0.362	0.347
δ(M|N_p_)	0.349	0.366	0.357	0.337	0.353	0.346
q(M|Cl)	0.779	0.793	0.763	0.757	0.770	0.740
δ(M|Cl)	0.536	0.564	0.552	0.574	0.606	0.588
q(M)	+2.171	+2.21	+2.125	+2.173	+2.218	+2.127
q(N_p_)	−1.191	−1.179	−1.187	−1.164	−1.153	−1.16
q(N_m_)	−1.121	−1.123	−1.121	−1.124	−1.125	−1.123
q(C_α_)	0.928	0.920	0.929	0.963	0.958	0.963
q(C_β_)	0.017	0.014	0.017	0.526	0.525	0.526
q(ligand) ^1^	−1.391	−1.423	−1.362	−1.416	−1.447	−1.386

^1^ The sum of the net atomic charges on the ligand atoms.

**Table 3:** Calculated mposition of the lowest excited states and corresponding oscillator strengths for MClPz and MClTTDPz complexes.

State	Composition (%)	Λ (nm)	f	
YClPz				
1^1^ E	$2a_{1}\to e^{*}$ (16) $a_{2}\to e^{*}$ (82)	510	0.15	λ_1_
4^1^ E	$1a_{1}\to e^{*}$ (53) $2a_{1}\to e^{*}$ (38) $a_{2}\to e^{*}$ (5)	334	0.13	λ_2_
5^1^ E	$1a_{1}\to e^{*}$ (41) $2a_{1}\to e^{*}$ (42) $a_{2}\to e^{*}$ (10)	312	0.60	λ_3_
LaClPz				
1^1^ E	$3a_{1}\to e^{*}$ (15) $a_{2}\to e^{*}$ (82)	510	0.15	λ_1_
4^1^ E	$2a_{1}\to e^{*}$ (47) $3a_{1}\to e^{*}$ (44) $a_{2}\to e^{*}$ (4)	346	0.08	λ_2_
6^1^ E	$1a_{1}\to e^{*}\;$(12) $2a_{1}\to e^{*}$ (38) $3a_{1}\to e^{*}$ (36) $a_{2}\to e^{*}$ (11)	311	0.62	λ_3_
LuClPz				
1^1^E	$2a_{1}\to e^{*}$ (16) $a_{2}\to e^{*}$ (82)	510	0.16	λ_1_
4^1^E	$1a_{1}\to e^{*}$ (56) $2a_{1}\to e^{*}$ (35) $a_{2}\to e^{*}$ (5)	332	0.12	λ_2_
5^1^E	$1a_{1}\to e^{*}$ (39) $2a_{1}\to e^{*}$ (45) $a_{2}\to e^{*}$ (11)	311	0.62	λ_3_
YClTTDPz				
1^1^ E	$5a_{1}\to 1e^{*}$ (5) $3a_{2}\to 1e^{*}$ (91)	588	0.27	λ_1_
5^1^ E	$4a_{1}\to 1e^{*}$ (43) $5a_{1}\to 1e^{*}$ (45) $2b_{1}\to 1e^{*}$ (10)	342	0.23	λ_2_
6^1^ E	$3a_{1}\to 1e^{*}$ (5) $4a_{1}\to 1e^{*}$ (41) $5a_{1}\to 1e^{*}$ (36) $3a_{2}\to 1e^{*}$ (6) $3a_{2}\to 2e^{*}$ (7)	319	0.67	λ_3_
LaClTTDPz				
1^1^ E	$5a_{1}\to 1e^{*}$ (6) $3a_{2}\to 1e^{*}$ (91)	582	0.27	λ_1_
5^1^ E	$4a_{1}\to 1e^{*}$ (80) $5a_{1}\to 1e^{*}$ (10) $3b_{1}\to 1e^{*}$ (10)	344	0.08	
6^1^ E	$3a_{1}\to 1e^{*}$ (21) $4a_{1}\to 1e^{*}$ (7) $5a_{1}\to 1e^{*}$ (59) $3a_{2}\to 1e^{*}$ (5) $3a_{2}\to 2e^{*}$ (6)	329	0.48	λ_2_
7^1^ E	$2a_{1}\to 1e^{*}$ (13) 3$a_{1}\to 1e^{*}$ (61) $5a_{1}\to 1e^{*}$ (14)	308	0.36	λ_3_
LuClTTDPz				
1^1^ E	$5a_{1}\to 1e^{*}$ (5) $3a_{2}\to 1e^{*}$ (91)	590	0.27	λ_1_
5^1^ E	$4a_{1}\to 1e^{*}$ (39) $5a_{1}\to 1e^{*}$ (49) $3b_{1}\to 1e^{*}$ (10)	342	0.26	λ_2_
6^1^ E	$3a_{1}\to 1e^{*}$ (5) $4a_{1}\to 1e^{*}$ (45) $5a_{1}\to 1e^{*}$ (32) $3a_{2}\to 1e^{*}$ (6) $3a_{2}\to 2e^{*}$ (7)	318	0.67	λ_3_

**Table 4:** Assignment of the IR vibrations of the MClPz and MClTTDPz complexes.

Frequency, cm^−1^	I_rel_, %	Symmetry	Assignment ^1^	Exp, cm^−1^
YClTTDPz				
1101.6(ω_80_-ω_81_)	59	E	r(N_p_-C_α_) (24), r(N_m_-C_α_) (28), r(C_α_-C_β_) (15), r(C_β_-N_t_) (9), φ(C_β_C_β_N_t_) (6), φ(C_β_N_t_S) (7)	1090 acac [20]
1280.0(ω_87_-ω_88_)	100	E	r(N_p_-C_α_) (27), r(C_β_-C_β_) (10), r(C_β_-N_t_) (10), φ(C_α_N_p_C_α_) (6), φ(N_p_C_α_N_m_) (10), φ(N_m_C_α_C_β_) (9), φ(C_α_C_β_N_t_) (6)	1260 acac [20]
LaClTTDPz				
1100.2(ω_80_-ω_81_)	61	E	r(N_p_-C_α_) (28), r(N_m_-C_α_) (27), r(C_α_-C_β_) (14), r(C_β_-N_t_) (8), φ(C_β_C_β_N_t_) (5), φ(C_β_N_t_S) (6)	
1274.8(ω_87_-ω_88_)	100	E	r(N_p_-C_α_) (29), r(C_β_-C_β_) (8), r(C_β_-N_t_) (9), φ(C_α_N_p_C_α_) (6), φ(N_p_C_α_N_m_) (10), φ(C_α_N_m_C_α_) (5), φ(N_m_C_α_C_β_) (9), φ(C_α_C_β_N_t_) (6)	
LuClTTDPz				
1102.1(ω_80_-ω_81_)	59	E	r(N_p_-C_α_) (24), r(N_m_-C_α_) (28), r(C_α_-C_β_) (15), r(C_β_-N_t_) (9), φ(C_β_C_β_N_t_) (6), φ(C_β_N_t_S) (7)	1090 acac [20]
1281.5(ω_87_-ω_88_)	100	E	r(N_p_-C_α_) (26), r(C_β_-C_β_) (10), r(C_β_-N_t_) (10), φ(C_α_N_p_C_α_) (6), φ(N_p_C_α_N_m_) (10), φ(N_m_C_α_C_β_) (9), φ(C_α_C_β_N_t_) (8)	1262 acac [20]
YClPz				
839.81 (ω_48_)	46	A_1_	OPB(C_β_-N_p_-N_m_-C_α_) (35), OPB(H-C_α_-C_β_-C_β_) (44), θ(C_α_-N_m_-C_α_-N_p_) (5), θ(N_m_-C_α_-C_β_-C_β_) (11)	
982.30 (ω_53_-ω_54_)	100	E	r(N_p_-Y) (5), r(N_p_-C_α_) (14), r(C_α_-C_β_) (53), r(C_α_-C_β_) (5)	
LaClPz				
838.13 (ω_48_)	44	A_1_	OPB(C_β_-N_p_-N_m_-C_α_) (35), OPB(H-C_α_-C_β_-C_β_) (44), θ(C_α_-N_m_-C_α_-N_p_) (5), θ(N_m_-C_α_-C_β_-C_β_) (11)	
975.65(ω_53_-ω_54_)	100	E	r(N_p_-La) (5), r(N_p_-C_α_) (15), r(N_m_-C_α_) (5), r(C_α_-C_β_) (56)	
LuClPz				
839.62 (ω_48_)	46	A_1_	OPB(C_β_-N_p_-N_m_-C_α_) (35), OPB(H-C_α_-C_β_-C_β_) (45), θ(C_α_-N_m_-C_α_-N_p_) (5), θ(N_m_-C_α_-C_β_-C_β_) (11)	
983.45 (ω_53_-ω_54_)	100	E	r(N_p_-Lu) (5), r(N_p_-C_α_) (7), r(N_p_-C_α_) (7), r(C_α_-C_β_) (26), r(C_α_-C_β_) (27)	

^1^ Coordinates are listed provided that their contributions (shown in parentheses) are greater than ~5%. Assignment of vibrational modes based on potential energy distribution. The following designations of the coordinates are used: r—stretching of the bond; φ—bending, a change in the angle; OPB—out-of-plane bending; θ—a change in the dihedral angle.

## Data Availability

The data presented in this study are available in supplementary material.

## Supplementary Materials

Cartesian coordinates of YClPz optimized B3LYP/pcseg-2 level of theory, Cartesian coordinates of LaClPz optimized B3LYP/pcseg-2 level of theory, Cartesian coordinates of LuClPz optimized B3LYP/pcseg-2 level of theory, Cartesian coordinates of YClTTDPz optimized B3LYP/pcseg-2 level of theory, Cartesian coordinates of LaClTTDPz optimized B3LYP/pcseg-2 level of theory, Cartesian coordinates of LuClTTDPz optimized B3LYP/pcseg-2 level of theory, Table S1: Calculated composition of the lowest excited states and corresponding oscillator strengths for MClPz and MClTTDPz complexes, Table S2: Assignment of the IR vibrations of the MClPz and MClTTDPz complexes, Table S3: Bond lengths and topological parameters of ρ(r) in bond critical points of the MClPz, Table S4: Bond lengths and topological parameters of ρ(r) in bond critical points of the MClTTDPz, Table S5: Charge on atoms in MClPz and MClTTDPz.
