# Supplementary material for: DFT Study of Molecular and Electronic Structure of Y, La and Lu Complexes with Porphyrazine and Tetrakis(1,2,5-thiadiazole)porphyrazine
Source: Molecules. 2020 Dec 29;26(1):113. doi: 10.3390/molecules26010113 (PMC7795284; doi:10.3390/molecules26010113)
Supplement: Supplementary file 1 [file molecules-26-00113-s001.pdf]

Supporting information for

«DFT Study of Molecular and Electronic Structure of Y, La and Lu Complexes with Porphyrazine and tetrakis(1,2,5-thiadiazole)porphyrazine»

Yuriy A. Zhabanov, Igor V. Ryzhov, Ilya A. Kuzmin, Alexey V. Eroshin and Pavel A. Stuzhin

Ivanovo State University of Chemistry and Technology, Research Institute of Chemistry of Macroheterocyclic Compounds, Sheremetievskiy av. 7, 153000 Ivanovo, Russian Federation

Content

Cartesian coordinates of YClPz optimized B3LYP/pcseg-2 level of theory: ... 2

Cartesian coordinates of LaClPz optimized B3LYP/pcseg-2 level of theory: ... 2

Cartesian coordinates of LuClPz optimized B3LYP/pcseg-2 level of theory: ... 3

Cartesian coordinates of YClTTDPz optimized B3LYP/pcseg-2 level of theory: ... 4

Cartesian coordinates of LaClTTDPz optimized B3LYP/pcseg-2 level of theory: ... 4

Cartesian coordinates of LuClTTDPz optimized B3LYP/pcseg-2 level of theory: ... 5

Table S1. Bond lengths and topological parameters of ρ(r) in bond critical points of the MClPz. ... 7

Table S2. Bond lengths and topological parameters of ρ(r) in bond critical points of the MClTTDPz. ... 8

Table S3. Charge on atoms in MClPz and MClTTDPz ... 9

Cartesian coordinates of YClPz optimized B3LYP/pcseg-2 level of theory:

N	0.000000000000	1.993336000000	-0.255275000000
N	1.993336000000	0.000000000000	-0.255275000000
N	0.000000000000	-1.993336000000	-0.255275000000
N	-1.993336000000	0.000000000000	-0.255275000000
N	2.371598000000	2.371598000000	-0.544869000000
N	2.371598000000	-2.371598000000	-0.544869000000
N	-2.371598000000	-2.371598000000	-0.544869000000
N	-2.371598000000	2.371598000000	-0.544869000000
C	1.104829000000	2.768875000000	-0.485831000000
C	2.768875000000	-1.104829000000	-0.485831000000
C	-1.104829000000	-2.768875000000	-0.485831000000
C	2.768875000000	1.104829000000	-0.485831000000
C	1.104829000000	-2.768875000000	-0.485831000000
C	-2.768875000000	-1.104829000000	-0.485831000000
C	-1.104829000000	2.768875000000	-0.485831000000
C	-2.768875000000	1.104829000000	-0.485831000000
C	0.677262000000	4.124663000000	-0.789624000000
C	4.124663000000	-0.677262000000	-0.789624000000
C	-0.677262000000	-4.124663000000	-0.789624000000
C	4.124663000000	0.677262000000	-0.789624000000
C	0.677262000000	-4.124663000000	-0.789624000000
C	-4.124663000000	-0.677262000000	-0.789624000000
C	-0.677262000000	4.124663000000	-0.789624000000
C	-4.124663000000	0.677262000000	-0.789624000000
H	1.353490000000	4.932188000000	-1.013263000000
H	4.932188000000	-1.353490000000	-1.013263000000
H	-1.353490000000	-4.932188000000	-1.013263000000
H	4.932188000000	1.353490000000	-1.013263000000
H	1.353490000000	-4.932188000000	-1.013263000000
H	-4.932188000000	-1.353490000000	-1.013263000000
H	-1.353490000000	4.932188000000	-1.013263000000
H	-4.932188000000	1.353490000000	-1.013263000000
Y	0.000000000000	0.000000000000	0.872219000000
Cl	0.000000000000	0.000000000000	3.395025000000

Cartesian coordinates of LaClPz optimized B3LYP/pcseg-2 level of theory:

N	0.000000000000	2.009718000000	-0.388711000000
N	2.009718000000	0.000000000000	-0.388711000000
N	0.000000000000	-2.009718000000	-0.388711000000
N	-2.009718000000	0.000000000000	-0.388711000000
N	2.369737000000	2.369737000000	-0.727586000000
N	2.369737000000	-2.369737000000	-0.727586000000
N	-2.369737000000	-2.369737000000	-0.727586000000
N	-2.369737000000	2.369737000000	-0.727586000000
C	1.103590000000	2.771593000000	-0.661221000000
C	2.771593000000	-1.103590000000	-0.661221000000
C	-1.103590000000	-2.771593000000	-0.661221000000
C	2.771593000000	1.103590000000	-0.661221000000
C	1.103590000000	-2.771593000000	-0.661221000000
C	-2.771593000000	-1.103590000000	-0.661221000000
C	-1.103590000000	2.771593000000	-0.661221000000
C	-2.771593000000	1.103590000000	-0.661221000000
C	0.677092000000	4.115201000000	-1.022975000000

C	4.115201000000	-0.677092000000	-1.022975000000
C	-0.677092000000	-4.115201000000	-1.022975000000
C	4.115201000000	0.677092000000	-1.022975000000
C	0.677092000000	-4.115201000000	-1.022975000000
C	-4.115201000000	-0.677092000000	-1.022975000000
C	-0.677092000000	4.115201000000	-1.022975000000
C	-4.115201000000	0.677092000000	-1.022975000000
H	1.353375000000	4.909759000000	-1.289221000000
H	4.909759000000	-1.353375000000	-1.289221000000
H	-1.353375000000	-4.909759000000	-1.289221000000
H	4.909759000000	1.353375000000	-1.289221000000
H	1.353375000000	-4.909759000000	-1.289221000000
H	-4.909759000000	-1.353375000000	-1.289221000000
H	-1.353375000000	4.909759000000	-1.289221000000
H	-4.909759000000	1.353375000000	-1.289221000000
La	0.000000000000	0.000000000000	1.031468000000
Cl	0.000000000000	0.000000000000	3.742224000000

Cartesian coordinates of LuClPz optimized B3LYP/pcseg-2 level of theory:

N	0.000000000000	1.990100000000	-0.354695000000
N	1.990100000000	0.000000000000	-0.354695000000
N	0.000000000000	-1.990100000000	-0.354695000000
N	-1.990100000000	0.000000000000	-0.354695000000
N	2.372115000000	2.372115000000	-0.632046000000
N	2.372115000000	-2.372115000000	-0.632046000000
N	-2.372115000000	-2.372115000000	-0.632046000000
N	-2.372115000000	2.372115000000	-0.632046000000
C	1.105186000000	2.768593000000	-0.574884000000
C	2.768593000000	-1.105186000000	-0.574884000000
C	-1.105186000000	-2.768593000000	-0.574884000000
C	2.768593000000	1.105186000000	-0.574884000000
C	1.105186000000	-2.768593000000	-0.574884000000
C	-2.768593000000	-1.105186000000	-0.574884000000
C	-1.105186000000	2.768593000000	-0.574884000000
C	-2.768593000000	1.105186000000	-0.574884000000
C	0.677306000000	4.126877000000	-0.865184000000
C	4.126877000000	-0.677306000000	-0.865184000000
C	-0.677306000000	-4.126877000000	-0.865184000000
C	4.126877000000	0.677306000000	-0.865184000000
C	0.677306000000	-4.126877000000	-0.865184000000
C	-4.126877000000	-0.677306000000	-0.865184000000
C	-0.677306000000	4.126877000000	-0.865184000000
C	-4.126877000000	0.677306000000	-0.865184000000
H	1.353440000000	4.937114000000	-1.078960000000
H	4.937114000000	-1.353440000000	-1.078960000000
H	-1.353440000000	-4.937114000000	-1.078960000000
H	4.937114000000	1.353440000000	-1.078960000000
H	1.353440000000	-4.937114000000	-1.078960000000
H	-4.937114000000	-1.353440000000	-1.078960000000
H	-1.353440000000	4.937114000000	-1.078960000000
H	-4.937114000000	1.353440000000	-1.078960000000
Lu	0.000000000000	0.000000000000	0.716089000000
Cl	0.000000000000	0.000000000000	3.208314000000

Cartesian coordinates of YClTTDPz optimized B3LYP/pcseg-2 level of theory:

N	0.000000000000	2.051700000000	0.058178000000
N	-2.051700000000	0.000000000000	0.058178000000
N	0.000000000000	-2.051700000000	0.058178000000
N	2.051700000000	0.000000000000	0.058178000000
N	-2.390747000000	2.390747000000	-0.156747000000
N	-2.390747000000	-2.390747000000	-0.156747000000
N	2.390747000000	-2.390747000000	-0.156747000000
N	2.390747000000	2.390747000000	-0.156747000000
C	1.139914000000	2.806802000000	-0.117198000000
C	-2.806802000000	1.139914000000	-0.117198000000
C	-1.139914000000	-2.806802000000	-0.117198000000
C	-2.806802000000	-1.139914000000	-0.117198000000
C	1.139914000000	-2.806802000000	-0.117198000000
C	2.806802000000	1.139914000000	-0.117198000000
C	-1.139914000000	2.806802000000	-0.117198000000
C	2.806802000000	-1.139914000000	-0.117198000000
C	-0.710892000000	4.181178000000	-0.346152000000
C	-4.181178000000	-0.710892000000	-0.346152000000
C	0.710892000000	-4.181178000000	-0.346152000000
C	-4.181178000000	0.710892000000	-0.346152000000
C	-0.710892000000	-4.181178000000	-0.346152000000
C	4.181178000000	-0.710892000000	-0.346152000000
C	0.710892000000	4.181178000000	-0.346152000000
C	4.181178000000	0.710892000000	-0.346152000000
N	1.261767000000	5.355725000000	-0.570618000000
N	-5.355725000000	1.261767000000	-0.570618000000
N	-1.261767000000	-5.355725000000	-0.570618000000
N	-5.355725000000	-1.261767000000	-0.570618000000
N	1.261767000000	-5.355725000000	-0.570618000000
N	5.355725000000	1.261767000000	-0.570618000000
N	-1.261767000000	5.355725000000	-0.570618000000
N	5.355725000000	-1.261767000000	-0.570618000000
S	0.000000000000	6.389954000000	-0.759840000000
S	-6.389954000000	0.000000000000	-0.759840000000
S	0.000000000000	-6.389954000000	-0.759840000000
S	6.389954000000	0.000000000000	-0.759840000000
Y	0.000000000000	0.000000000000	1.127579000000
Cl	0.000000000000	0.000000000000	3.624089000000

Cartesian coordinates of LaClTTDPz optimized B3LYP/pcseg-2 level of theory:

N	0.000000000000	2.068301000000	0.011564000000
N	-2.068301000000	0.000000000000	0.011564000000
N	0.000000000000	-2.068301000000	0.011564000000
N	2.068301000000	0.000000000000	0.011564000000
N	-2.389442000000	2.389442000000	-0.251569000000
N	-2.389442000000	-2.389442000000	-0.251569000000
N	2.389442000000	-2.389442000000	-0.251569000000
N	2.389442000000	2.389442000000	-0.251569000000
C	1.139075000000	2.810705000000	-0.206125000000
C	-2.810705000000	1.139075000000	-0.206125000000
C	-1.139075000000	-2.810705000000	-0.206125000000
C	-2.810705000000	-1.139075000000	-0.206125000000

C	1.139075000000	-2.810705000000	-0.206125000000
C	2.810705000000	1.139075000000	-0.206125000000
C	-1.139075000000	2.810705000000	-0.206125000000
C	2.810705000000	-1.139075000000	-0.206125000000
C	-0.711225000000	4.176340000000	-0.493377000000
C	-4.176340000000	-0.711225000000	-0.493377000000
C	0.711225000000	-4.176340000000	-0.493377000000
C	-4.176340000000	0.711225000000	-0.493377000000
C	-0.711225000000	-4.176340000000	-0.493377000000
C	4.176340000000	-0.711225000000	-0.493377000000
C	0.711225000000	4.176340000000	-0.493377000000
C	4.176340000000	0.711225000000	-0.493377000000
N	1.261874000000	5.338538000000	-0.773498000000
N	-5.338538000000	1.261874000000	-0.773498000000
N	-1.261874000000	-5.338538000000	-0.773498000000
N	-5.338538000000	-1.261874000000	-0.773498000000
N	1.261874000000	-5.338538000000	-0.773498000000
N	5.338538000000	1.261874000000	-0.773498000000
N	-1.261874000000	5.338538000000	-0.773498000000
N	5.338538000000	-1.261874000000	-0.773498000000
S	0.000000000000	6.363860000000	-1.010597000000
S	-6.363860000000	0.000000000000	-1.010597000000
S	0.000000000000	-6.363860000000	-1.010597000000
S	6.363860000000	0.000000000000	-1.010597000000
La	0.000000000000	0.000000000000	1.388401000000
Cl	0.000000000000	0.000000000000	4.067734000000

Cartesian coordinates of LuClTTDPz optimized B3LYP/pcseg-2 level of theory:

N	0.000000000000	2.048312000000	-0.045052000000
N	-2.048312000000	0.000000000000	-0.045052000000
N	0.000000000000	-2.048312000000	-0.045052000000
N	2.048312000000	0.000000000000	-0.045052000000
N	-2.391057000000	2.391057000000	-0.248271000000
N	-2.391057000000	-2.391057000000	-0.248271000000
N	2.391057000000	-2.391057000000	-0.248271000000
N	2.391057000000	2.391057000000	-0.248271000000
C	1.140162000000	2.806151000000	-0.210129000000
C	-2.806151000000	1.140162000000	-0.210129000000
C	-1.140162000000	-2.806151000000	-0.210129000000
C	-2.806151000000	-1.140162000000	-0.210129000000
C	1.140162000000	-2.806151000000	-0.210129000000
C	2.806151000000	1.140162000000	-0.210129000000
C	-1.140162000000	2.806151000000	-0.210129000000
C	2.806151000000	-1.140162000000	-0.210129000000
C	-0.710843000000	4.182099000000	-0.426204000000
C	-4.182099000000	-0.710843000000	-0.426204000000
C	0.710843000000	-4.182099000000	-0.426204000000
C	-4.182099000000	0.710843000000	-0.426204000000
C	-0.710843000000	-4.182099000000	-0.426204000000
C	4.182099000000	-0.710843000000	-0.426204000000
C	0.710843000000	4.182099000000	-0.426204000000
C	4.182099000000	0.710843000000	-0.426204000000
N	1.261746000000	5.358993000000	-0.638377000000
N	-5.358993000000	1.261746000000	-0.638377000000
N	-1.261746000000	-5.358993000000	-0.638377000000

N	-5.358993000000	-1.261746000000	-0.638377000000
N	1.261746000000	-5.358993000000	-0.638377000000
N	5.358993000000	1.261746000000	-0.638377000000
N	-1.261746000000	5.358993000000	-0.638377000000
N	5.358993000000	-1.261746000000	-0.638377000000
S	0.000000000000	6.394917000000	-0.817002000000
S	-6.394917000000	0.000000000000	-0.817002000000
S	0.000000000000	-6.394917000000	-0.817002000000
S	6.394917000000	0.000000000000	-0.817002000000
Lu	0.000000000000	0.000000000000	0.964277000000
Cl	0.000000000000	0.000000000000	3.431209000000

Table S1. Bond lengths and topological parameters of ρ(r) in bond critical points of the MClPz.

Interaction	r_e (Å)	ρ (a.u.)	$\nabla^2\rho$ (a.u.)	λ_1	λ_2	λ_3	ε	G_b(a.u.)	V_b(a.u.)	H_b(a.u.)	$q(A\vert B)$	$\delta(A\vert B)$
**YClPz**												
**r(N$_m$-C$_\alpha$)**	1.329	0.357	-1.263	-0.865	-0.751	0.353	0.151	0.227	-0.771	0.543	0.560	1.243
**r(N$_p$-C$_\alpha$)**	1.370	0.331	-1.056	-0.771	-0.681	0.396	0.132	0.175	-0.614	0.439	0.421	1.131
**r(Np-Y)**	2.291	0.069	0.205	-0.098	-0.083	0.385	0.180	0.062	-0.073	0.011	0.348	0.349
**r(La–Cl)**	2.523	0.060	0.170	-0.058	-0.058	0.286	0.000	0.053	-0.064	0.011	0.779	0.536
**r(C–H)**	1.077	0.297	-1.228	-0.838	-0.828	0.438	0.012	0.041	-0.389	0.348	0.038	0.964
**r(C$_\beta$–C$_\beta$)**	1.355	0.341	-0.993	-0.766	-0.612	0.385	0.252	0.130	-0.508	0.378	0.000	1.594
**r(C$_\alpha$–C$_\beta$)**	1.454	0.288	-0.774	-0.621	-0.545	0.391	0.140	0.080	-0.353	0.273	0.054	1.068
**LaClPz**												
**r(N$_m$-C$_\alpha$)**	1.330	0.355	-1.254	-0.861	-0.747	0.353	0.151	0.226	-0.766	0.540	0.562	1.242
**r(N$_p$-C$_\alpha$)**	1.369	0.332	-1.059	-0.774	-0.683	0.398	0.133	0.175	-0.616	0.440	0.412	1.137
**r(N$_p$-La)**	2.461	0.063	0.160	-0.078	-0.066	0.304	0.182	0.049	-0.059	0.010	0.356	0.366
**r(La–Cl)**	2.711	0.054	0.135	-0.045	-0.045	0.225	0.000	0.042	-0.050	0.008	0.793	0.564
**r(C–H)**	1.077	0.297	-1.226	-0.837	-0.827	0.438	0.013	0.041	-0.388	0.347	0.036	0.964
**r(C$_\beta$–C$_\beta$)**	1.354	0.341	-0.993	-0.766	-0.612	0.385	0.253	0.130	-0.509	0.378	0.000	1.597
**r(C$_\alpha$–C$_\beta$)**	1.456	0.287	-0.768	-0.617	-0.542	0.391	0.140	0.079	-0.351	0.271	0.050	1.065
**LuClPz**												
**r(N$_m$-C$_\alpha$)**	1.329	0.357	-1.265	-0.866	-0.752	0.353	0.151	0.228	-0.772	0.544	0.560	1.244
**r(N$_p$-C$_\alpha$)**	1.370	0.330	-1.055	-0.771	-0.681	0.397	0.132	0.175	-0.614	0.439	0.423	1.128
**r(Np-Lu)**	2.260	0.075	0.235	-0.108	-0.093	0.436	0.158	0.060	-0.071	0.011	0.340	0.357
**r(La–Cl)**	2.493	0.065	0.187	-0.065	-0.065	0.316	0.000	0.051	-0.063	0.012	0.763	0.552
**r(C–H)**	1.077	0.297	-1.228	-0.838	-0.828	0.438	0.012	0.041	-0.389	0.348	0.038	0.964
**r(C$_\beta$–C$_\beta$)**	1.355	0.341	-0.992	-0.765	-0.612	0.385	0.251	0.130	-0.508	0.378	0.000	1.593
**r(C$_\alpha$–C$_\beta$)**	1.454	0.289	-0.775	-0.621	-0.545	0.392	0.140	0.080	-0.354	0.274	0.055	1.069

Table S2. Bond lengths and topological parameters of ρ(r) in bond critical points of the MClTTDPz.

Interaction	r_e (Å)	ρ (a.u.)	$\nabla^2\rho$ (a.u.)	λ_1	λ_2	λ_3	ε	G_b(a.u.)	V_b(a.u.)	H_b(a.u.)	q(A|B)	δ(A|B)
**YClTTDPz**												
***r*(N_m-C_α)**	1.319	0.361	-1.176	-0.840	-0.724	0.388	0.160	0.295	-0.883	0.589	0.522	1.259
***r*(N_t–C_β)**	1.317	0.361	-1.176	-0.840	-0.724	0.388	0.160	0.295	-0.883	0.589	0.522	1.350
***r*(N_p-C_α)**	1.379	0.323	-1.000	-0.747	-0.652	0.399	0.147	0.172	-0.593	0.422	0.405	1.130
***r*(Np-La)**	2.313	0.066	0.193	-0.092	-0.079	0.364	0.173	0.058	-0.068	0.010	0.354	0.337
***r*(Y–Cl)**	2.497	0.064	0.176	-0.063	-0.063	0.301	0.000	0.057	-0.070	0.013	0.757	0.574
***r*(C_β–C_β)**	1.422	0.312	-0.866	-0.691	-0.589	0.414	0.174	0.096	-0.408	0.312	0.000	1.093
***r*(C_α–C_β)**	1.458	0.286	-0.768	-0.619	-0.540	0.391	0.145	0.076	-0.344	0.268	0.004	1.008
***r*(N_t-S)**	1.643	0.235	-0.342	-0.375	-0.277	0.310	0.355	0.180	-0.445	0.265	0.615	1.308
**LaClTTDPz**												
***r*(N_m-C_α)**	1.320	0.361	-1.277	-0.877	-0.749	0.350	0.171	0.242	-0.804	0.561	0.562	1.258
***r*(N_t–C_β)**	1.316	0.361	-1.176	-0.841	-0.724	0.389	0.161	0.296	-0.886	0.590	0.526	1.352
***r*(N_p-C_α)**	1.377	0.324	-1.006	-0.751	-0.654	0.400	0.148	0.173	-0.597	0.424	0.395	1.137
***r*(Np-La)**	2.486	0.061	0.152	-0.074	-0.063	0.289	0.179	0.047	-0.056	0.009	0.362	0.353
***r*(La–Cl)**	2.680	0.058	0.140	-0.049	-0.049	0.238	0.000	0.045	-0.055	0.010	0.770	0.606
***r*(C_β–C_β)**	1.423	0.311	-0.864	-0.690	-0.588	0.414	0.174	0.095	-0.407	0.311	0.000	1.092
***r*(C_α–C_β)**	1.460	0.284	-0.762	-0.615	-0.537	0.390	0.145	0.075	-0.342	0.266	0.001	1.006
***r*(N_t-S)**	1.644	0.235	-0.344	-0.375	-0.276	0.307	0.355	0.179	-0.443	0.264	0.611	1.307
**LuClTTDPz**												
***r*(N_m-C_α)**	1.319	0.363	-1.289	-0.884	-0.755	0.350	0.170	0.245	-0.811	0.567	0.562	1.260
***r*(N_t–C_β)**	1.317	0.361	-1.176	-0.840	-0.724	0.387	0.160	0.294	-0.883	0.588	0.521	1.349
***r*(N_p-C_α)**	1.379	0.323	-0.998	-0.747	-0.651	0.400	0.147	0.171	-0.592	0.421	0.406	1.128
***r*(N_p-Lu)**	2.284	0.072	0.221	-0.102	-0.088	0.411	0.152	0.056	-0.066	0.010	0.347	0.346
***r*(La–Cl)**	2.468	0.069	0.195	-0.070	-0.070	0.334	0.000	0.054	-0.068	0.014	0.740	0.588
***r*(C_β–C_β)**	1.422	0.312	-0.866	-0.691	-0.589	0.414	0.174	0.096	-0.408	0.312	0.000	1.093
***r*(C_α–C_β)**	1.458	0.286	-0.770	-0.620	-0.541	0.391	0.145	0.076	-0.344	0.268	0.005	1.008
***r*(N_t-S)**	1.643	0.235	-0.342	-0.376	-0.277	0.310	0.355	0.180	-0.445	0.265	0.615	1.308

Table S3. Charge on atoms in MClPz and MClTTDPz

YClPz

Name	C_β	C_α	H	N_p	N_m	Y	Cl	On ligand
q(A)	+0.0166	+0.928	+0.038	-1.191	-1.121	+2.171	-0.779	-1.391

LaClPz

Name	C_β	C_α	H	N_p	N_m	La	Cl	On ligand
q(A)	+0.14	+0.92	+0.36	-1.179	-1.123	+2.21	-0.793	-1.423

LuClPz

Name	C_β	C_α	H	N_p	N_m	Lu	Cl	On ligand
q(A)	+0.017	+0.929	+0.038	-1.187	-1.121	+2.125	-0.763	-1.362

YClTTDPz

Name	C_β	C_α	N_p	N_m	Y	Cl	S	N_t	On ligand
q(A)	+0.526	+0.963	-1.164	-1.124	+2.173	-0.757	+1.23	-1.137	-1.416

LaClTTDPz

Name	C_β	C_α	N_p	N_m	La	Cl	S	N_t	On ligand
q(A)	+0.525	+0.958	-1.153	-1.125	+2.218	-0.77	+1.223	-1.137	-1.447

LuClTTDPz

Name	C_β	C_α	N_p	N_m	Lu	Cl	S	N_t	On ligand
q(A)	+0.526	+0.963	-1.16	-1.123	+2.127	-0.74	+1.231	-1.137	-1.386
